# Migrating longitudinal African mental health data from staging to the OMOP common data model within the INSPIRE network datahub

**DOI:** 10.3389/fpsyt.2026.1751529

**Published:** 2026-03-09

**Authors:** Tathagata Bhattacharjee, Bylhah Mugotitsa, Michael Ochola, Reinpeter Momanyi, Pauline Andeso, David Amadi, Dorothy Mailosi, Letisha Najjemba, Jay Greenfield, Kagiso Mabe, Emma Slaymaker, Jim Todd, Agnes Kiragga, INSPIRE Network

**Affiliations:** 1 London School of Hygiene and Tropical Medicine (LSHTM), London, United Kingdom; 2 African Population and Health Research Center (APHRC), Nairobi, Kenya; 3 Strathmore University Business School, Strathmore University, Nairobi, Kenya; 4 CODATA-Committee on Data of the International Science Council, Paris, France; 5 Department of Information and Knowledge Management, University of Johannesburg, Johannesburg, South Africa; 6 Catholic University of Health and Allied Sciences (CUHAS), Mwanza, Tanzania; 7 Infectious Diseases Institute, College of Health Sciences, Makerere University, Kampala, Uganda

**Keywords:** Africa, data harmonization, data quality assurance, health informatics, longitudinal studies, mental disorders, mental Health, OHDSI

## Abstract

**Background:**

The standardization and integration of longitudinal mental health data from African cohort studies are critical in advancing research and informing policy. There are several challenges posed by diverse sources, instruments adapted for locals, and the absence of an interoperable framework to allow for meaningful analysis and cross-study comparisons.

**Methods:**

We designed and executed a metadata-driven pipeline using the OMOP Common Data Model within the INSPIRE Network Datahub to harmonise multi-country African mental health datasets. Data extracted previously from longitudinal studies, standardised via a snowflake schema staging database, is now mapped to OMOP vocabularies with local extensions, and validated through quality assurance protocols using OHDSI tools.

**Results:**

A total of 202,013 person records and over 7 million observations across fourteen cohort studies were successfully migrated. Mapping completeness exceeded 99.9%, with high conformance, completeness, and plausibility across all OMOP domains. Custom vocabularies ensured the coverage of context-specific exposures and outcomes, thereby supporting robust cohort construction, event characterization, and longitudinal analyses.

**Conclusion:**

This framework demonstrates scalable harmonisation and integration of African mental health data, bridging the gap between local datasets with global standards. This then enables the performance of federated analysis and reproducible research, increasing the utility and impact of mental health data in informing evidence-based policies and future collaborative studies across Africa.

## 1 Introduction

This paper continues the effort to standardise mental health data in Africa. It builds on the previous work of mapping source mental health surveillance data, collected using different questionnaires, to a Staging database, as discussed in the paper published by 1. The method used the Data Documentation Initiative (DDI) Lifecycle and Observational Medical Outcomes Partnership (OMOP) vocabularies within the INSPIRE Network Datahub (1). The initial work tackled the significant challenges of managing diverse mental health datasets from different African studies. It organised these datasets in a metadata-rich, snowflake schema-based staging database, specifically designed for population health analysis. A list of local concepts was developed to address gaps in the OMOP vocabulary, allowing the outcomes of mental health surveillance to be meaningfully represented and integrated into the staging database design.

The mental health burden in African countries is increasing. Socioeconomic gaps, stigma, and limited healthcare services make issues like depression, anxiety, and psychosis worse (2, October 12). In the past, healthcare data efforts in Africa have focused on infectious diseases, which has led to a lack of standardised mental health data collection (3, 4, December 6). There is a need to track long-term trends, use various data collection tools, and consider changing population dynamics (5). This creates more demands on data systems. Capturing the temporal and multicentric complexities of longitudinal mental health studies, including dynamic exposures and social determinants, requires robust data architectures capable of integrating diverse information sources (6, 7). Atewologun et al. (8) provide a comprehensive review of mental health services in sub-Saharan Africa, highlighting progress and persistent challenges. Therefore, harmonising the mental health data into a globally accepted standard for research and analysis is critical.

Developments in the standardisation of population health data in Africa have created the necessary foundation for this study. The INSPIRE datahub project introduced a range of integrated services for the harmonisation of longitudinal health datasets across Africa using the OMOP Common Data Model and OHDSI tools to enhance data interoperability and analytic capabilities (9). In addition, a successful mapping of COVID-19 sero-surveillance data from the Nairobi Urban Health and Demographic Surveillance System to the OMOP CDM exhibited an example of effective data transformation methods in a real-world population health setting (10). Taken together, these foundational studies established best practices and technical processes for harmonisation, and are the basis for this current work that builds upon these methods for longitudinal African mental health research for improved regional and global impact.

The FAIR (Findable, Accessible, Interoperable, Reusable) principles provide a widely adopted framework for improving the reuse of scientific data, particularly in distributed and heterogeneous research environments (11).

Improvements in metadata standards (e.g., DDI-Lifecycle) allow for the systematic documentation and semantic annotation of survey instruments, variables, and response values, improving interoperability and reusability of mental health datasets (12). Building upon this work, recent and ongoing efforts demonstrate that making metadata machine-readable is a key first step to implementing the FAIR principles—findable, accessible, interoperable, and reusable—for population health data. By creating frameworks to make metadata machine-readable and interoperable, researchers increased discoverability, integration, and analytic readiness of complex health datasets, thus facilitating scalable harmonisation and reuse in global health settings (13).

Moving beyond psychiatry-specific projects, several major initiatives have developed methodological frameworks to retrospectively harmonise data from diverse study cohorts. Fortier et al. (14) established a set of best practices and Fortier et al. (15) built on this to prioritise transformations guided by detailed metadata and emphasize the importance of transparent documentation. More recently, Cheng et al. (16) illustrated a scalable pipeline approach for harmonizing longitudinal, real-world datasets. Together, these studies underscore the significant technical and governance hurdles involved in harmonizing human subject data after its collection. The INSPIRE Mental Health staging database follows these standards within a snowflake schema. It captures the timing and multi-center complexities of long-term health studies, including changing exposures and social factors linked to mental health outcomes (1). This kind of semantic alignment supports the change of various datasets into standardised, machine-readable formats. As a result, it greatly improves secondary research capabilities.

The Observational Health Data Sciences and Informatics (OHDSI) collaborative has developed a comprehensive open-science ecosystem for observational data standardization and analytics, including the OMOP Common Data Model (CDM), ATLAS for cohort definition, and ACHILLES for data quality characterization (17, 18). Transforming staging datasets into the OMOP CDM improves data sharing, FAIRification, and analysis, a process that benefits from the global OHDSI community and its collection of tools for defining, characterising, and analysing cohorts (19) to support comparative research across regions. However, African mental health datasets face unique challenges, such as locally developed codes, concepts, and exposures. To address this, custom vocabularies and extension methods are needed to support standard OMOP terminology (20, 21).

This paper discusses the design, implementation, and evaluation of an ETL (Extract, Transform, Load) pipeline that supports the migration from the INSPIRE Mental Health staging database to the OMOP CDM in Africa’s mental health research. It emphasises mapping local mental health concepts to OMOP vocabularies, checking data quality, and allowing improved analysis with OHDSI tools like ACHILLES, ATLAS, and the OHDSI GIS extension, through which spatiotemporal analyses of MH events and outcomes are supported. This work builds on the previous INSPIRE effort and shows a scalable, interoperable model for standardising mental health data across Africa. It encourages a data-driven approach to mental health policy and intervention.

## 2 Method

### 2.1 Source dataset overview

The source datasets comprise longitudinal mental health data collected from multiple population-based cohort studies across Africa. These studies capture rich, repeated measures of mental health outcomes over time.

#### 2.1.1 Longitudinal mental health datasets considered for migration

The longitudinal mental health datasets for this research were extracted from various African population-based studies, incorporating both primary and secondary data collected across multiple waves at individual and household levels. Through multiple Extraction, Transformation, and Loading (ETL) processes, these datasets were harmonised within a staging database structured with a snowflake schema to capture complex temporal relationships, and documented using international standards and semantic annotations (OMOP and local vocabularies) to ensure quality and readiness for mapping to the OMOP Common Data Model (CDM). **Table 1** summarizes these diverse studies from Kenya, Ethiopia, Uganda, and South Africa, which investigate outcomes such as depression, anxiety, PTSD, sleep, and pain among different adult populations, including clinic attendees and HDSS cohorts. These studies encompass a range of covariates—sociodemographics, health behaviors, maternal factors, religious involvement, and environmental influences—revealing the multidimensional landscape of mental health across African contexts using robust longitudinal and panel methods.

**Table 1:** African mental health cohort studies summary.

Study ID	Country	Study description	Phenotype group	Primary focus	Covariates	Version date	DOI
1	Kenya	Postpartum Depression	Antepartum women in health care clinic	Postpartum depression	Demographics, Risk factors, Postnatal risk factors	2018-10-01	10.1186/s12888-018-1904-7
2	Ethiopia	PTSD Trajectories	Postpartum Women	Trajectories of PTSD symptoms and mediating relationships	Socio-demographics, maternal morbidities, obstetric variables, health-related quality of life, psychosocial factors	2022-04-11	10.1371/journal.pone.0266399
3	Ethiopia	Postnatal Multimorbidity	Postpartum Women	Postnatal comorbid and multimorbid anxiety, depression, PTSD	Socio-demographics, maternal morbidities, obstetric variables, health-related quality of life, psychosocial factors	2022-08-15	10.1371/journal.pone.0273176
4	South Africa	Intrusive Pain and Mental Health	Participants with intrusive pain	Risk factors for incident/persistent intrusive pain	Sociodemographics, substance use, and physical activity	2022-04-26	10.1515/sjpain-2022-0013
5	South Africa	Sleep and Mental Health	Rural adults 40+	Sleep duration (short/long) and correlates	Sociodemographics, health behaviors	2018-06-28	10.3390/ijerph15071357
6	South Africa	Negative Household events & Depression vulnerability	Adults in South Africa	Negative household events and depressive symptoms	Sociodemographic and economic factors, living conditions	2017-04-25	10.1016/j.jad.2017.04.031
7	South Africa	Depression and poverty	Individuals 15+	Poverty and depression cycles	Education, race, residence, income, employment	2018-03-15	10.1016/j.jad.2017.12.050
8	South Africa	Religion and depression risk	Individuals in South Africa	Religious activity and depressive symptoms risk	Religious involvement, sociodemographics	2018-01-05	10.1007/s10943-017-0551-5
9	South Africa	Smoking and Depression	Depression-free adults	Smoking and incident depression	Smoking, sociodemographics	2020-01-01	10.1093/ntr/nty163
10	South Africa	Clinic Proximity & Depression	Individuals in South Africa	Depressive symptoms risk, proximity to healthcare	Proximity to clinic, sociodemographics	2017-03-15	10.1007/s00127-017-1369-x
11	South Africa	Living Alone & Depression	Adults who live alone	Depression scores	Sociodemographics, residence, economic status, health conditions	2021-06-14	10.1016/j.ssmph.2021.100800
12	Uganda	Depression, Anxiety, and Psychosis in Iganga Mayuge HDSS	Adults at Iganga Mayuge-HDSS	Depression, Anxiety, Psychosis	Sociodemographics, mood & affective symptoms, anxiety & psychosomatic and behavioral	2024-05-15	Primary study data
13	Uganda	Depression, Anxiety, and Psychosis in Kagando HDSS	Adults at Kagando-HDSS	Depression, Anxiety, Psychosis	Sociodemographics, mood, psychotic symptoms, anxiety & stressand somatic	2024-02-01	Primary study data
14	Kenya	Depression, Anxiety, and Psychosis in Kilifi HDSS	Adults at Kilifi-HDSS	Depression, Anxiety, Psychosis	Sociodemographics, mood, psychotic symptoms, anxiety & stress and somatic	2024-07-01	Primary study data

In line with the distinction between publicly available standards and project-specific metadata, access to the individual-level data in this study is similarly governed by ethical and data use agreements. Due to these necessary restrictions associated with sensitive mental health information, the harmonised individual-level datasets cannot be deposited in public repositories. However, the methodological transparency is supported by the comprehensive metadata documentation, including our publicly available staging database framework. To improve compliance with FAIR data principles, we are exploring secure, controlled-access mechanisms via emerging platforms designed for sensitive human subject data.

#### 2.1.2 Instruments for mental health assessments

The mental health assessments were carried out using a selection of standardised psychometric instruments used globally in clinical and research settings, and where applicable, were adapted for the African contexts. The instruments are listed with a brief description in **Table 2**.

**Table 2:** Mental health assessment instruments summary.

Instrument Number	Instrument name	Description
i	Perceived Stress Questionnaire (PSQ)	A 30-item self-report measure that captures perceived stress experiences subjectively, including dimensions such as harassment, overload, irritability, lack of enjoyment, fatigue, worry, and tension. The PSQ reflects cognitive perceptions about stress that have occurred over specific time periods, which can range from recent times to longer periods. Given its high internal consistency (Cronbach’s alpha ~0.9) and good test-retest reliability, it has good psychometric properties and has been validated with various student and diverse psychiatric, healthcare worker, and healthy adult populations, making it appropriate for examining general community mental health.
ii	Center for Epidemiologic Studies Depression Scale (CES-D)	Assesses depressive symptomatology in the general population, focusing on the frequency of depressive symptoms experienced in the past week.
iii	PTSD Checklist for DSM-5 (PCL-5)	A 20-item self-report measure for screening and symptom monitoring of post-traumatic stress disorder according to DSM-5 criteria.
iv	Edinburgh Postnatal Depression Scale (EPDS)	A 10-item screening tool specifically designed to identify postnatal depression in new mothers.
V	Depression, Anxiety, and Stress Scales (DASS-21)	A shortened version of the original DASS, this instrument measures three related negative emotional states, namely depression, anxiety, and stress, providing a quick, reliable way to assess emotional distress.
vi	Generalized Anxiety Disorder-7 (GAD-7)	A brief 7-item scale commonly used to screen for and measure the severity of generalized anxiety disorder.
vii	Patient Health Questionnaire-5 (PHQ-5)	A short form of the PHQ, designed to screen for depression and anxiety symptoms efficiently in population health surveys.

Taken together, these tools provide a comprehensive and multidimensional measure of mental health, measuring stress, depression, anxiety, trauma symptoms, and postpartum mental health issues. These tools were used in longitudinal cohort studies in Kenya, Ethiopia, South Africa, and Uganda, with published papers enabling monitoring of mental health trajectories in diverse African populations.

#### 2.1.3 Staging database design and metadata schema

The staging database serves as the central, metadata-rich repository for harmonising heterogeneous longitudinal mental health datasets before their migration into the OMOP Common Data Model (CDM). As described in our foundational work (1), the database is architected using a snowflake schema designed to capture the complexity of longitudinal population studies. This design is optimized to represent households, individuals, data collection waves, and research instruments, and to preserve the temporal relationships among them.

Interoperability in the staging layer is supported by the Data Documentation Initiative (DDI) Lifecycle standard (13). The DDI-aligned metadata provide structured descriptions of each contributing study, including study design, sampling approach, instrument content, variable definitions, response categories, and value labels. This level of annotation preserves provenance, supports reproducibility, and maintains the contextual meaning of the original African mental health data across sites.

Study metadata, including variable definitions, value domains, and collection protocols, were curated centrally within the INSPIRE project to support harmonisation across sites. Due to the sensitive nature of mental health data and site-specific ethical and governance restrictions, the metadata are not publicly released but are available under controlled access for bona fide research purposes.

### 2.2 Staging database overview

The staging database serves as an intermediate, standardised repository harmonising multi-source longitudinal mental health data using a snowflake schema and rich metadata, before migration into the OMOP CDM. Due to site-specific governance and consent constraints, the harmonisation process resulted in the creation of 14 separate, site-specific OMOP CDM instances rather than a single consolidated database, resulting in a federated analysis system.

#### 2.2.1 Semantic annotation using DDI-lifecycle and vocabulary development (schema.org)

The semantic annotation, guided by the DDI Lifecycle model, aligns key staging database entities (household, individual, wave, instrument, variables) to DDI elements, ensuring semantic consistency, provenance, and interoperability across integrated mental health studies. By combining DDI-Lifecycle with schema.org-based JSON-LD, the framework supports machine-readable, web-discoverable datasets, advancing FAIR principles and preparing harmonised mental health data for transformation into the OMOP CDM and use within the OHDSI ecosystem.

#### 2.2.2 Local concepts extension to OMOP vocabularies

To accommodate context-specific and culturally nuanced mental-health variables not represented in standard OMOP vocabularies, a localized extension, designated as the INSPIRE vocabulary, was developed. During the harmonization process, constructs unique to the African study settings, such as locally validated mental-health scales and site-specific response categories, lacked direct equivalents in existing standards like SNOMED or LOINC. To retain their analytical and semantic value, new concepts were created within OMOP’s vocabulary structure following Health Economics & Medical Informatics Standardisation (THEMIS) guidelines. Assigning local entries concept_ids above two billion (2,000,000,000) enables a clear distinction from global standards while preserving OMOP compatibility, with 323 concepts harmonised—44.9% from LOINC, 41.5% INSPIRE local, and 12.1% from SNOMED. This robust harmonisation is a significant achievement that warrants detailed documentation and broader application beyond INSPIRE Mental Health, as it is highly relevant for integrating data on other health conditions. This balanced integration demonstrates how the INSPIRE extension successfully preserved the semantic richness of study-specific data while ensuring interoperability within the OMOP Common Data Model. The localized vocabulary not only enables complete analytical coverage in ATLAS but also establishes a foundation for future contributions of validated African mental-health concepts to the broader OHDSI vocabulary ecosystem.

### 2.3 ETL pipeline architecture

The ETL (Extraction, Transformation, and Loading) pipeline is a key element of the migration process from the staging database to the OMOP Common Data Model. The pipeline facilitates the orderly extraction of longitudinal mental health data that has been standardised from within the staging environment, applies various transformation rules to the data to meet OMOP CDM standards and its associated vocabularies, and loads the consumed data in its transformed state into the OMOP CDM tables. The pipelined architecture is meant to preserve data integrity, retain temporal relationships, and support scalable and repeatable migration processes for varying data volume and originating data sources.

#### 2.3.1 ETL pipeline for staging-to-OMOP CDM migration

This study employed an Extract-Transform-Load (ETL) pipeline to migrate and harmonise mental health assessment data captured using validated instruments (PHQ-9, GAD-7, DASS-21, EPDS, PCL-5, CES-D, PSQ) from a staging database to the OMOP Common Data Model (CDM). The objective was to standardise data, preserve provenance, and enable semantic interoperability across population studies.

The pipeline was initiated by configuring the computational environment and the central working directory. Data were extracted from a relational PostgreSQL staging database, with study datasets stored in separate schemas. Secure connectivity ensured controlled access to cohort-specific records. Metadata tables were curated to define instrument structures, while demographic attributes were retained for traceability.

Within the target database, the OMOP-CDM schema and vocabulary structures were instantiated, and standardised vocabularies were loaded for semantic alignment. Responses to questions and composite scores were transformed into OMOP-compliant records. Post-migration, summary analytics evaluated completeness and harmonization, quality frameworks and dashboards supported systematic review, and analytic snapshots with metadata were archived for reproducibility and auditing.

**Figure 1:**
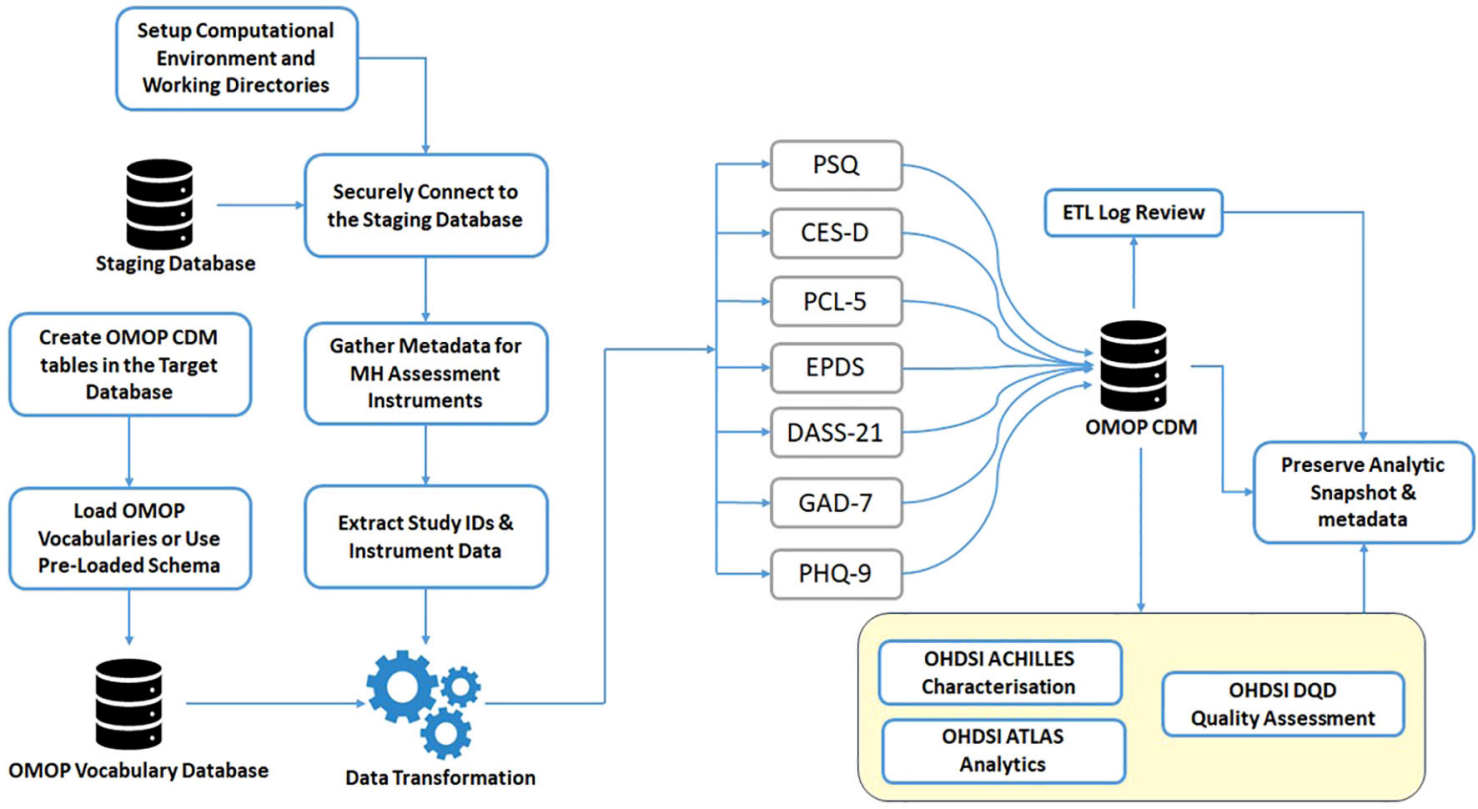
ETL pipeline for mental health data harmonisation from staging DB to OMOP CDM.

**Figure 1** shows the ETL pipeline that extracts, standardises, and semantically annotates mental health data from a staging database and maps it into the OMOP CDM for interoperable research use.

#### 2.3.2 ETL process using R for loading data into OMOP CDM from the staging database

**Figure 2:**
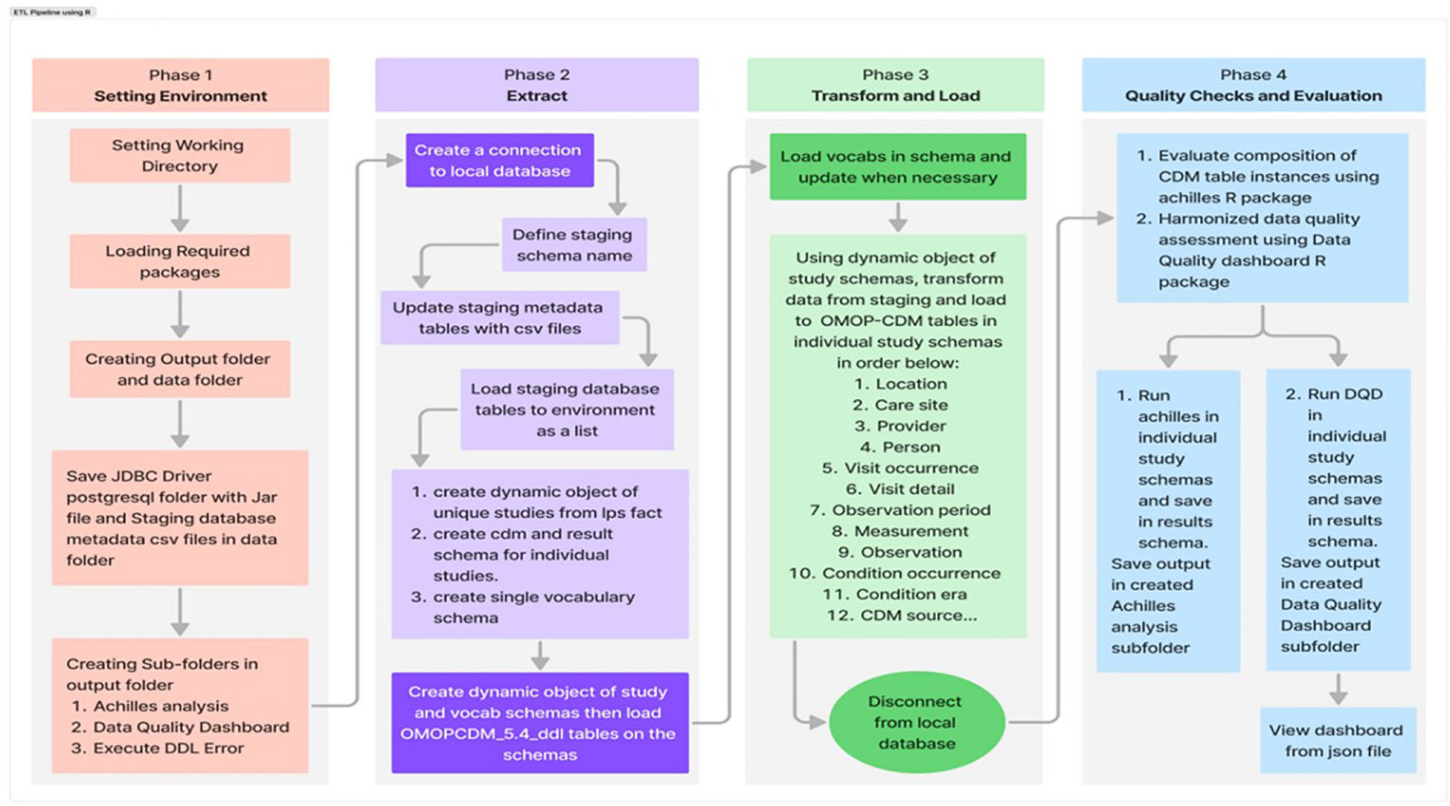
Implementation of ETL using R.

Using R, the ETL pipeline for loading data from the staging database to the OMOP CDM provides a structured, repeatable approach to performing high-quality ETL transformations. The ETL pipeline is a four-phase activity. Phase 1 establishes the R environment, loading the necessary packages, and creating appropriate data and output folders for important drivers and metadata files for downstream processes. Phase 2 includes connecting to the local database, defining the staging schema and vocabulary schema, refreshing the metadata file, and then dynamically loading the necessary staging tables to R based on the dynamic R objects. Phase 3 sequentially maps and loads data into the OMOP CDM tables in order (e.g. location, care site, providers, person, visit occurrence, etc.), and updates vocabulary terms and schemas before disconnecting from the database. Finally, Phase 4 performs quality checks using the Achilles R package and Data Quality Dashboard R package to effectively assess data quality, with results from the quality checks downloaded into appropriate data and output folders for dashboard use. This approach standardises and streamlines data integration, fostering interoperability. **Figure 2** shows the stepwise workflow of the key components used during the R-based ETL process to implement the OMOP CDM for mental health data, and the R scripts are available on GitHub (https://github.com/APHRC-DSE/INSPIRE-Mental-Health-Project-Integrating-and-Harmonizing-Longitudinal-Data).

The table order shown in Phase 3 of **Figure 2** reflects the practical execution order of the ETL pipeline based on table dependencies rather than the conceptual presentation order used in the Book of OHDSI.

### 2.4 Mapping local concepts to OMOP vocabulary

It is important to map the concepts of local source data to the OMOP common data model vocabulary to provide semantic interoperability and consistency across different datasets. The process involves automatically and manually mapping locally-specific codes and terminology to an OMOP standard concept so that data can be integrated and analysed reliably.

#### 2.4.1 The mapping framework for local mental health terms without OMOP equivalents

Local mental health terminologies that lacked standardized equivalents within the OMOP Common Data Model (CDM) vocabularies were identified and flagged for customization. Each term was reviewed and mapped to an appropriate OMOP domain (e.g., Measurement, Observation) based on its definition and context. These concepts were then assigned unique 2-billion-range concept identifiers(concept_ids ≥ 2,000,000,000) to prevent conflicts with existing OMOP standard concepts and to maintain consistency within the CDM structure.

#### 2.4.2 Procedure for creating custom vocabularies and integrating with OMOP

To ensure that locally defined concepts are visible and analyzable within ATLAS, the INSPIRE custom vocabularies were systematically integrated into the OMOP CDM vocabulary tables. These concepts were mapped as standard (S) to enable their use in analytical workflows and ATLAS-based cohort definitions. The main domains in which custom vocabularies were added were the Observation and Measurement. The vocabulary tables were populated in this way:

Step 1: Vocabulary table

A new entry defining the custom vocabulary was added, as shown in the **Table 3** below:

**Table 3:** Custom vocabulary.

Vocab_id	Vocab_name	Vocab_reference	Vocab_version	Vocab_concept_id
INSPIRE	Local Custom Vocabulary	https://github.com/APHRC-DSE/INSPIRE-Mental-Health-Project-Integrating-and-Harmonizing-Longitudinal-Data	v1 (First release for public access)	0

Step 2: Concept Table

Each locally defined term was added to the OMOP concept table under the designated domain, with the 2-billion range Concept IDs assigned, preventing overlap with existing OMOP concepts.

Step 3: Concept relationship table

Concept relationships were added to describe mappings such as Maps to, Maps from, Has answer, and Is an answer of. These relationships establish semantic links and interoperability between standard and custom concepts.

Step 4: Integrate custom concepts into their respective CDM tables

The newly created concepts were referenced in the corresponding fields within the OMOP CDM tables, specifically, in the Observation table (observation_concept_id, value_as_concept_id) and the Measurement table (measurement_concept_id) - to enable their inclusion in downstream analyses.

Although THEMIS conventions recommend placing non-standard concepts in the *_source_concept_id fields, this approach restricts ATLAS visualization of the custom concepts(REF - https://ohdsi.github.io/CommonDataModel/customConcepts.html). Therefore, to ensure both searchability and analytic usability within ATLAS, the custom vocabularies were included under the *_concept_id fields. All CSV files containing the custom vocabularies and associated mappings of the vocabulary tables are available in the INSPIRE project’s GitHub (https://github.com/APHRC-DSE/INSPIRE-Mental-Health-Project-Integrating-and-Harmonizing-Longitudinal-Data) repository here.

#### 2.4.3 Concept mapping strategy

Concept mapping was performed to align source variables with the OMOP Common Data Model and OHDSI standard vocabularies. Where equivalent standard concepts existed, variables were mapped directly to standard OHDSI concepts using the Athena vocabulary repository. Approximately 90% of variables were successfully mapped to standard concepts. Variables for which no suitable standard concept existed, most commonly study-specific questionnaire items and locally defined symptom measures, were represented using custom non-standard concepts. These custom concepts were systematically labeled with study-specific prefixes and documented to preserve semantic clarity.

Mappings were created primarily through manual expert review involving data scientists and domain experts, supported by OHDSI tooling. Variables that could not be meaningfully mapped were excluded from transformation to avoid semantic distortion. The inability to map certain concepts reflects limitations in existing vocabularies rather than data quality issues and highlights areas where future vocabulary extensions may be required.

The distinction between the OBSERVATION and MEASUREMENT tables followed OHDSI definitions. Quantitative variables with defined units or scales were mapped to MEASUREMENT, while qualitative assessments, symptom presence, and questionnaire responses without intrinsic units were mapped to OBSERVATION.

### 2.5 Data quality assurance and validation

The assurance of data quality during the ETL process is essential to the reliability of the OMOP CDM dataset. This section describes the validation steps that are incorporated into the ETL workflow, including statistical and semantic checks, and processes for managing missing or inconsistent data to preserve the trustworthiness and usability of the final dataset.

#### 2.5.1 Validation steps embedded within the ETL workflow

The INSPIRE Mental Health ETL pipeline embeds data quality validation immediately after loading into the OMOP CDM using ACHILLES and the Data Quality Dashboard (DQD). ACHILLES, an R package, generates ATLAS‐ready reports that produce descriptive statistics across all CDM tables to detect implausible value ranges, missing concepts, temporal inconsistencies, and outliers, guiding iterative ETL refinements. The DQD applies harmonised terminology to perform over 1,500 table‐ and field‐level checks organized by the Kahn framework across conformance, completeness, and plausibility dimensions. Each check compares violation rates against predefined thresholds, issuing PASS/FAIL outcomes, failure frequency summaries, and targeted visualisations to pinpoint curation needs. By embedding these quality checks in every ETL iteration, the pipeline achieves issue detection, feedback for script optimisation, and audit trails, ensuring the harmonised OMOP CDM meets the standards.

#### 2.5.2 Statistical and semantic validation checks

After loading data into the OMOP CDM, the R-based ETL workflow uses ACHILLES and DQD to perform statistical and semantic validation. The statistical aspects of the ACHILLES profile the distributions of data, frequency of clinical events, and temporal sequences of clinical events to identify potential anomalies and verify data integrity. The validation verifies concept mappings against standardised vocabularies, identifies source codes that are unmapped or deprecated, and confirms domain assignments for each concept and referential consistency across tables. Both statistical profiling and semantic validation rely on a series of SQL-based rules to generate detailed reports of violations, allowing data quality issues to be identified and corrected in the ETL process.

#### 2.5.3 Handling of missing or inconsistent data

The R-based ETL scripts implement a systematic approach to ensure data quality, employing conditional transformations and imputation strategies to handle missing or inconsistent data. Critical identifier fields (e.g., PERSON_ID, visit identifiers) are filtered using WHERE clauses to exclude NULL records, while semi-structured fields like gender and marital status are processed using COALESCE() functions within the dbExecute() SQL workflow. For numeric and date fields, CASE WHEN expressions validate logical consistency—for instance, flagging future birth dates or observation dates preceding birth dates, which are then recorded as NULL and logged via an INSERT INTO etl_error_log statement. Concept code consistency is ensured by joining incoming values against OMOP vocabulary tables; unmapped or deprecated codes are routed to review tables, while appropriate codes are remapped using a LEFT JOIN to internal mapping files imported via read_csv(). Throughout this process, pre- and post-processing row counts for each transformation step are recorded in an etl_execution_summary table, providing a comprehensive audit trail. During the staging and validation phases, null values in required fields such as person_id were observed due to incomplete source identifiers. Placeholder identifiers were deliberately not inserted to avoid introducing false referential integrity, and all final OMOP CDM tables conform to required field constraints.

### 2.6 Integration with OHDSI and other analytical tools

The standardised OMOP CDM format enables seamless integration with the OHDSI ecosystem and other analytical platforms, facilitating advanced longitudinal mental health research through shared tools and collaborative analyses.

#### 2.6.1 Integration and configuration of ATLAS pipelines

After data characterization and quality assessment checks were completed, the results schema (containing ACHILLES output tables), the CDM schema (containing standardized OMOP CDM tables), and the vocabulary schema were restored to a PostgreSQL environment hosted on AWS to support subsequent integration with ATLAS. The process of generating additional results tables and WebAPI configuration is documented in steps 2 and 3 respectively. The script produced in Step 2 created and populated the concept_hierarchy table within the results schema. This table serves as a lookup structure linking concepts across multiple domains (e.g., Condition, Drug, Drug Era, Measurement, Observation, and Procedure). Hierarchical relationships were derived from standardized vocabularies such as SNOMED CT, MedDRA, and ATC, using mappings defined in the concept and concept_ancestor tables within the vocabulary schema. The resulting structure enables ATLAS to visualize and navigate concept relationships, display treemaps, and support concept set design and cohort characterization. The incorporation of INSPIRE custom concepts into the vocabulary tables, as described in the previous step, ensured that both standardized OMOP CDM vocabularies and local INSPIRE extensions were visible and fully functional within ATLAS for analytical use.

#### 2.6.2 Integration with AutoML (No-Code/Low-Code)

The AutoML environment is designed as an all-in-one no-code/low-code framework that brings together every major stage of the analytics workflow under one interface. Within this environment, the OMOP Common Data Model (CDM) forms the foundation for data management and standardization. Once a user connects to the database, the AutoML system automatically recognizes and structures the data according to OMOP conventions, allowing the user to perform Data Quality Dashboard (DQD) checks, Achilles characterization, and OMOP data visualization. This ensures that datasets meet standard quality and consistency requirements before any analytical or modeling steps begin.

Beyond visualization and data quality, the AutoML platform integrates advanced OMOP functionalities such as cohort construction and feature extraction. Users can define study cohorts directly from OMOP tables, selecting populations based on standardized vocabularies and clinical concepts. After this, the feature extraction module compiles relevant variables from OMOP domains like person, condition occurrence, drug exposure, measurement, and observation, transforming them into a structured analysis-ready dataset.

Once feature extraction is complete, the resulting tables are automatically uploaded within the AutoML system, eliminating the need for manual data transfer. These extracted features are seamlessly passed to the machine-learning pipeline, where the Rautoml engine part of the APHRC No-Code App ecosystem (https://github.com/aphrc-nocode/no-code-app) handles the rest of the process. This includes automated data preprocessing, algorithm selection, hyper-parameter tuning, model evaluation, and result visualization.

By embedding OMOP-based data management, quality control, feature engineering, and AutoML modeling within one environment, the system provides a fully automated, reproducible, and accessible pipeline. Analysts can move from raw OMOP data to validated cohorts, extracted features, and trained predictive models without writing extensive code. This integration not only streamlines the analytical workflow but also supports scalability and reusability across health, climate, and education research applications.

## 3 Result

This section presents the key findings of the study, highlighting the insights gained from migrating the African mental health datasets to the OMOP CDM framework.

### 3.1 Data migration

Here, we discuss the successful migration of longitudinal African mental health datasets into the OMOP Common Data Model (CDM), highlighting the scope and completeness of the transferred data. Note that because the source studies captured in the staging database were not comparable in terms of the mental health conditions they screened and the factors the source studies observed that might have been related to these conditions, data from the staging database were not migrated into a single OMOP CDM instance. Instead, during data migration, fourteen (14) OMOP CDM datasets were created, one for each study. This changed the approach taken during analysis, as described below.

#### 3.1.1 Quantitative summary of records migrated

The dataset migration involved harmonizing records from the central staging database environment into the OMOP CDM structure. A total of 202,013 person records were successfully mapped. These 202,013 person records contributed 7,396,214 rows of information on the following:

- Visits storing information such as the date, location, and type of healthcare encounters.
- Measurements storing results from mental health assessments (e.g., PHQ-9 depression scores, GAD-7 anxiety scales), or other psychometric scales relevant to patient status, conditions, and observations.
- Condition storing diagnostic codes for mental health disorders like depression, anxiety, or substance use disorders, enabling standardized tracking across clinical and research settings.
- Observation storing self-reported symptoms, psychosocial variables (e.g., suicidal ideation, perceived stress), and responses to mental health surveys, providing context for behavioral analysis.

**Table 4** summarises the scale and distribution of harmonised mental health data across fourteen longitudinal studies following migration to the OMOP Common Data Model. Each row corresponds to a unique study and reports the number of person records, along with associated counts of key OMOP domains: visit occurrences, measurements, condition occurrences, and observations. Across all studies, the harmonisation process yielded a total of 202,013 person records, linked to 423,886 visits, 478,548 measurements (such as mental health scale scores), 161,695 condition occurrences (diagnoses), and a substantial 6,332,085 observations (behavioral, symptom, and survey responses). This aggregation highlights both the wide coverage and granularity of the integrated dataset, enabling comprehensive longitudinal analysis of mental health trends, conditions, and outcomes throughout diverse African populations.

**Table 4:** Quantitative summary of records migrated.

Study identifier	Person	Visit occurrence	Measurement	Condition occurrence	Observation
1	188	359	359	103	5503
2	775	2325	9300	1049	13950
3	775	2325	9300	1049	13950
4	5059	9235	14019	33356	215677
5	5059	5059	15177	17984	143969
6	25981	51609	50550	13944	733923
7	25981	51609	50550	13944	733923
8	25981	51609	50550	13944	733923
9	32060	74378	73304	19111	1045971
10	25981	51609	50550	13944	733923
11	37749	98278	97150	24592	1383928
12	9067	18134	35668	2569	374274
13	3171	3171	9513	2395	82788
14	4186	4186	12558	3711	116383
Total records	202013	423886	478548	161695	6332085

#### 3.1.2 Staging to OMOP CDM transformation rates and success metrics

The data transformation from the central staging database to the OMOP CDM demonstrated exceptionally high mapping completeness across all domains: Person, Visit Occurrence, Measurement, Condition Occurrence, and Observation, with overall mapping rates consistently above 99.9%. Overall, the ETL process achieved near-complete data fidelity, with negligible losses during transformation. The minimal exclusions observed were justified by OMOP CDM THEMIS mapping convention protocols that prioritize data completeness and analytic reliability for age-dependent variables. These results indicate a robust and reliable ETL process, successfully operationalizing the OMOP CDM for downstream analytic use.

**Table 5** presents transformation rates and success metrics for harmonising mental health data into the OMOP CDM across fourteen studies. For each OMOP domain, like the person, visit occurrence, measurement, condition occurrence, and observation, the % of data successfully mapped is shown alongside any exclusions. The results demonstrate exceptionally high mapping fidelity, with at least 99.9% of records in each category successfully transformed and very few exclusions during the process. Most studies achieved a perfect 100% mapping rate, while a small number had minor exclusions, but overall integrity was maintained. These metrics highlight the effectiveness and robustness of the harmonization workflow, ensuring comprehensive and reliable representation of longitudinal mental health data.

**Table 5:** Transformation rates and success metrics.

Study ID	Person		Visit occurrence		Measurement		Condition occurrence		Observation	
	Excluded in mapping	% Mapped	Excluded during mapping	% Mapped	Excluded during mapping	% Mapped	Excluded during mapping	% Mapped	Excluded during mapping	% Mapped
1	0	100.00%	0	100.00%	0	100.00%	0	100.00%	0	100.00%
2	0	100.00%	0	100.00%	0	100.00%	0	100.00%	0	100.00%
3	0	100.00%	0	100.00%	0	100.00%	0	100.00%	0	100.00%
4	0	100.00%	0	100.00%	0	100.00%	0	100.00%	0	100.00%
5	0	100.00%	0	100.00%	0	100.00%	0	100.00%	0	100.00%
6	22	99.92%	23	99.96%	22	99.96%	13	99.91%	320	99.96%
7	22	99.92%	23	99.96%	22	99.96%	13	99.91%	320	99.96%
8	22	99.92%	23	99.96%	22	99.96%	13	99.91%	320	99.96%
9	23	99.93%	26	99.97%	25	99.97%	13	99.93%	356	99.97%
10	22	99.92%	23	99.96%	22	99.96%	13	99.91%	320	99.96%
11	24	99.94%	27	99.97%	26	99.97%	13	99.95%	367	99.97%
12	0	100.00%	0	100.00%	0	100.00%	0	100.00%	0	100.00%
13	0	100.00%	0	100.00%	0	100.00%	0	100.00%	0	100.00%
14	2	99.95%	2	99.95%	6	99.95%	0	100.00%	52	99.96%
Total records	137	99.93%	147	99.97%	145	99.97%	78	99.95%	2055	99.97%

### 3.2 Vocabulary integration outcomes

This section summarises the results of mapping local source terminologies to OMOP standardised vocabularies, highlighting coverage, gaps, and semantic consistency achieved.

#### 3.2.1 Local concepts successfully mapped to OMOP vocabularies

Although the vocabulary harmonization process demonstrated strong standardization coverage, we could not map all source terms to OMOP vocabularies. Instead of dropping the unmapped records, we created local custom concepts by following the THEMIS set conventions and incorporated them successfully into the concept table. This ensured all source terms would be reliably used in downstream analytic pipelines.

#### 3.2.2 Implementation of custom vocabulary and filling coverage gaps

A total of 255 unique concepts were identified across OMOP domains. Of these, 227 (89.0%) were successfully mapped to standard OMOP vocabularies, while 28 (11.0%) remained as local concepts pending curation or inclusion in standardised terminologies.

**Figure 3:**
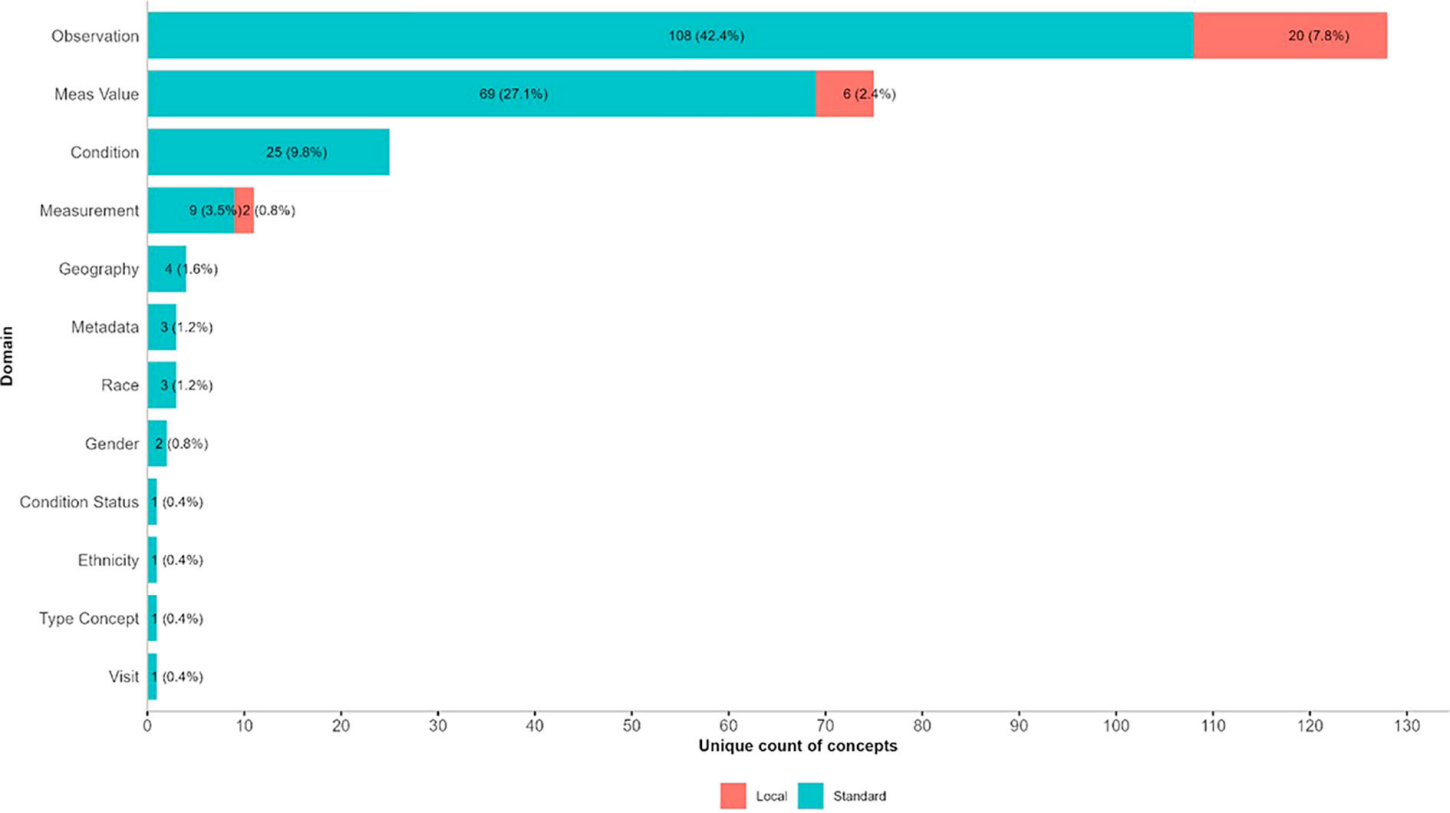
Distribution of standard and local concepts by OMOP domain.

**Figure 3** illustrates the distribution of standard and local concepts assigned across OMOP domains in the harmonised mental health dataset. Most concepts are standard OMOP vocabulary entries, within the Observation, Meas Value, and Condition domains, suggesting a successful mapping to global terminologies and facilitating interoperability. local concepts, shown in red, appear alongside standard ones in the domains of Observation (20 local concepts), Meas Value (6), and Measurement (2), indicating the necessity for custom entries to capture region-specific or non-standardised elements unique to African mental health research. The remaining domains (Geography, Metadata, Race, Gender, Condition Status, Ethnicity, Type Concept, and Visit) have very few concepts overall, with almost all being standard. This figure highlights both the completeness of OMOP mapping and the pragmatic use of local extensions for semantic fidelity, ensuring comprehensive and context-relevant representation of mental health data.

#### 3.2.3 Critical mental health concepts unique to African contexts

The vocabulary harmonisation achieved a high standardization rate (89%). The remaining local concepts were primarily associated with custom site-specific observations and non-standard measurement values or units.

Observation domains represented the largest share of unique concepts, accounting for 42.4% of all standardised concepts. The high proportion of standardised mappings reflects robust integration of observational data elements, such as clinical assessments, behavioral factors, and social determinants. However, the residual local concepts (7.8%) highlight heterogeneity in site-specific observation capture.

The Measurement domain contributed 4.3% of the concepts (3.5% standard, 0.8% local), while Measurement Value (Meas Value) accounted for 29.5% of the concepts (27.1% standard, 2.4% local). These were primarily instrument total scores and result category measurements, showing good alignment with LOINC and OMOP measurement concepts. The remaining local values represent units or qualitative result categories that have not yet been standardised.

Comprising 9.8% standard concepts, the Condition domain achieved full standardisation coverage within SNOMED-based OMOP condition concepts. This reflects a strong mapping of diagnosis and clinical condition data.

Demographic and Administrative domains, such as Gender, Race, Geography, and Metadata, were fully mapped to OMOP standard concepts supporting consistency in data provenance and site-level representation. The Ethnicity, Condition Status, Type Concept, and Visit domains each contributed one standardized concept, underscoring the low variability and completeness of their mapping.

### 3.3 Data quality and validation results

Data quality assessment was conducted using the OHDSI Data Quality Dashboard (DQD) Version 2.6.3 implemented in R. The DQD systematically evaluated conformance (compliance with specifications of field types, mandatory fields, and key constraints), completeness (missingness across key domains), plausibility (logical and temporal relationships), and vocabulary integrity (valid concept IDs and standard concept mappings) across all OMOP CDM tables and fields.

#### 3.3.1 Results of data quality checks

The OHDSI Data Quality Dashboard (DQD) provided a comprehensive evaluation of mapping performance, running thousands of checks across completeness, plausibility, and conformance domains. Overall, studies achieved a 98% pass rate, with completeness and plausibility reaching 100%—indicating no missing data or implausible values—and conformance checks attaining 94–97%, mainly affected by minor structural issues such as intentionally blank cohort tables. Good completeness means all fields are correctly populated, with NULL values accepted when allowed by the OMOP CDM; plausibility reflects values within logical ranges; and conformance ensures consistent adherence to OMOP table structures and terminology. Records that fail these checks are flagged for review, allowing for correction, exclusion, or annotation rather than wholesale rejection, with acceptance thresholds set by project policy. **Table 6** summarizes data quality metrics across all site-specific OMOP CDM instances, reinforcing the high standards achieved in this study. The near-perfect pass rates across all studies are reflected in the identical values displayed across columns, a result of applying uniform validation rules and consistent transformation logic throughout the harmonization pipeline. This consistency demonstrates the stability and reproducibility of the process, ultimately confirming the robustness and reliability of the harmonized mental health dataset for advanced OMOP-based research and analysis.

**Table 6:** Results of data quality checks.

Study identifier	Data quality checks	Verification				Validation				Total			
		Pass	Fail	Total	% Pass	Pass	Fail	Total	% Pass	Pass	Fail	Total	% Pass
1	Plausibility	516	4	520	99%	291	0	291	100%	807	4	811	100%
	Conformance	866	23	889	97%	133	8	141	94%	999	31	1030	97%
	Completeness	452	0	452	100%	17	0	17	100%	469	0	469	100%
	Total	1834	27	1861	99%	441	8	449	98%	2275	35	2310	98%
2	Plausibility	516	4	520	99%	291	0	291	100%	807	4	811	100%
	Conformance	866	23	889	97%	133	8	141	94%	999	31	1030	97%
	Completeness	452	0	452	100%	17	0	17	100%	469	0	469	100%
	Total	1834	27	1861	99%	441	8	449	98%	2275	35	2310	98%
3	Plausibility	516	4	520	99%	291	0	291	100%	807	4	811	100%
	Conformance	866	23	889	97%	133	8	141	94%	999	31	1030	97%
	Completeness	452	0	452	100%	17	0	17	100%	469	0	469	100%
	Total	1834	27	1861	99%	441	8	449	98%	2275	35	2310	98%
4	Plausibility	516	4	520	99%	291	0	291	100%	807	4	811	100%
	Conformance	866	23	889	97%	133	8	141	94%	999	31	1030	97%
	Completeness	452	0	452	100%	17	0	17	100%	469	0	469	100%
	Total	1834	27	1861	99%	441	8	449	98%	2275	35	2310	98%
5	Plausibility	516	4	520	99%	291	0	291	100%	807	4	811	100%
	Conformance	866	23	889	97%	133	8	141	94%	999	31	1030	97%
	Completeness	452	0	452	100%	17	0	17	100%	469	0	469	100%
	Total	1834	27	1861	99%	441	8	449	98%	2275	35	2310	98%
6	Plausibility	516	4	520	99%	291	0	291	100%	807	4	811	100%
	Conformance	866	23	889	97%	133	8	141	94%	999	31	1030	97%
	Completeness	452	0	452	100%	17	0	17	100%	469	0	469	100%
	Total	1834	27	1861	99%	441	8	449	98%	2275	35	2310	98%
7	Plausibility	516	4	520	99%	291	0	291	100%	807	4	811	100%
	Conformance	866	23	889	97%	133	8	141	94%	999	31	1030	97%
	Completeness	452	0	452	100%	17	0	17	100%	469	0	469	100%
	Total	1834	27	1861	99%	441	8	449	98%	2275	35	2310	98%
8	Plausibility	516	4	520	99%	291	0	291	100%	807	4	811	100%
	Conformance	866	23	889	97%	133	8	141	94%	999	31	1030	97%
	Completeness	452	0	452	100%	17	0	17	100%	469	0	469	100%
	Total	1834	27	1861	99%	441	8	449	98%	2275	35	2310	98%
9	Plausibility	516	4	520	99%	291	0	291	100%	807	4	811	100%
	Conformance	866	23	889	97%	133	8	141	94%	999	31	1030	97%
	Completeness	452	0	452	100%	17	0	17	100%	469	0	469	100%
	Total	1834	27	1861	99%	441	8	449	98%	2275	35	2310	98%
10	Plausibility	516	4	520	99%	291	0	291	100%	807	4	811	100%
	Conformance	866	23	889	97%	133	8	141	94%	999	31	1030	97%
	Completeness	452	0	452	100%	17	0	17	100%	469	0	469	100%
	Total	1834	27	1861	99%	441	8	449	98%	2275	35	2310	98%
11	Plausibility	516	4	520	99%	291	0	291	100%	807	4	811	100%
	Conformance	866	23	889	97%	133	8	141	94%	999	31	1030	97%
	Completeness	452	0	452	100%	17	0	17	100%	469	0	469	100%
	Total	1834	27	1861	99%	441	8	449	98%	2275	35	2310	98%
12	Plausibility	516	4	520	99%	291	0	291	100%	807	4	811	100%
	Conformance	866	23	889	97%	133	8	141	94%	999	31	1030	97%
	Completeness	452	0	452	100%	17	0	17	100%	469	0	469	100%
	Total	1834	27	1861	99%	441	8	449	98%	2275	35	2310	98%
13	Plausibility	516	4	520	99%	291	0	291	100%	807	4	811	100%
	Conformance	866	23	889	97%	133	8	141	94%	999	31	1030	97%
	Completeness	452	0	452	100%	17	0	17	100%	469	0	469	100%
	Total	1834	27	1861	99%	441	8	449	98%	2275	35	2310	98%
14	Plausibility	516	4	520	99%	291	0	291	100%	807	4	811	100%
	Conformance	866	23	889	97%	133	8	141	94%	999	31	1030	97%
	Completeness	452	0	452	100%	17	0	17	100%	469	0	469	100%
	Total	1834	27	1861	99%	441	8	449	98%	2275	35	2310	98%

### 3.4 OHDSI analytical outputs

This section highlights the primary analytical results generated using OHDSI tools, demonstrating the capacity of the OHDSI toolset to conduct a meta-analysis using OHDSI cohort construction, cohort characterisation, incidence rate analyses, and path analysis across the succession of mental health conditions.

#### 3.4.1 General approach

The general approach was to conduct a meta-analysis across the fourteen (14) OMOP CDM datasets using OHDSI tools. To this end, the following steps were taken:

- We created cohorts across all the studies based on the results of screening and diagnostic instruments like GAD-7 and PHQ-9.
- We characterised each cohort in each study demographically and, depending on the study, by socioeconomic status, work history, and, with women, parity and gravidity.
- Again, depending on the study, we calculated incidence rates for mental health outcomes with various “target cohorts” or, again, populations like women who had multiple births over time, men with a history of unemployment, people with a religious affiliation, and so forth.
- We created “event cohorts” for each mental health condition, depending on the study, and conducted study-specific pathway analyses that observed the succession of these event cohorts.

#### 3.4.2 More specifically

In ATLAS, descriptive analysis tools like cohort characterisation and incidence analysis begin with cohort construction. Given this methodology, cohorts for anxiety disorder and depression disorder that could be constructed across all the studies were developed. The table below summarises the criteria and methodology used for cohort construction in ATLAS, enabling cross-study descriptive analyses of anxiety and depression disorder populations.

**Table 7** presents the distinct number of people and total number of episodes for each study, with both values being identical because the cohort definition was restricted to the earliest event for each participant. This means that only the first qualifying episode was counted per person, preventing multiple episodes from the same individual being included. The result is a clear, one-to-one mapping between individuals and events, which simplifies cohort analysis, reduces bias from repeated measures, and ensures consistency in evaluating initial mental health outcomes across diverse studies and populations.

**Table 7:** Summary of cohort construction and outcome counts across studies.

Study ID	Study code	Distinct number of people	Total number of episodes
1	01_study_Kenya	85	85
2	02_study_Ethiopia	150	150
3	03_study_Ethiopia	150	150
4	04_study_HAALSI	2,119	2,119
5	05_study_HAALSI	837	837
6	06_study_NIDS	11,131	11,131
7	07_study_NIDS	11,131	11,131
8	08_study_NIDS	11,131	11,131
9	09_study_NIDS	14,236	14,236
10	10_study_NIDS	11,131	11,131
11	11_study_NIDS	17,219	17,219
12	12_study_Iganga	1,296	1,296
13	13_study_Kagando	629	629
14	14_study_Kilifi	1,040	1,040

### 3.5 Cohort characterisation by study

In this system, constructing a cohort refers to creating a subset of people from the larger dataset based on specific criteria (such as having an anxiety disorder, depression disorder, or any mental health condition). This process involves extracting and manipulating data to form distinct groups for further analysis, not just tracking longitudinal groups as in traditional epidemiology.

Once these cohorts are defined, a characterisation using a descriptive analysis is performed for each group in each study. The ATLAS tool enables this by organising information into different “domain” tables (e.g., demographics, conditions, measurements). Within each table, users can select preset variables, and ATLAS automatically runs univariate descriptive statistics (counts, means, proportions, etc.) for each selected feature. For example, in Study #1 (study ID = 1), after constructing each cohort, users can review demographic summaries (such as age, sex, geographic location) based on these presets.

**Figure 4:**
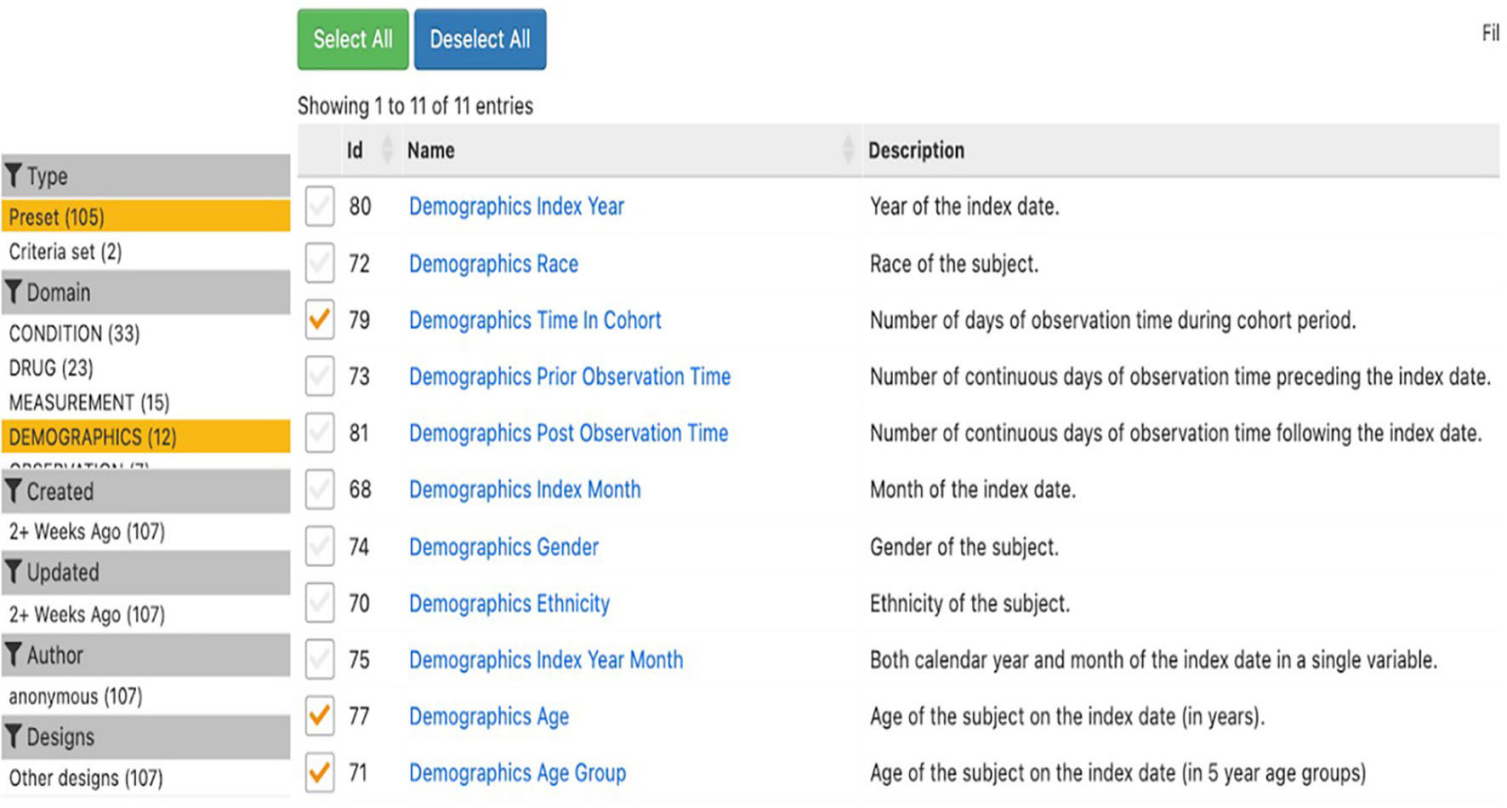
Demographic and observation variables available for cohort characterisation in ATLAS.

**Figure 4** displays the range of demographic and observation variables available for cohort characterisation in the ATLAS. Users can select demographic presets, such as index year, race, gender, age, age group, and ethnicity, alongside observation time features like prior and post observation duration. These options allow for detailed descriptive summaries of cohort populations, facilitating analysis of temporal coverage, age distributions, and key population characteristics at the time of cohort entry. The figure demonstrates the flexibility and granularity offered by ATLAS, supporting robust demographic profiling essential for mental health and epidemiological research.

**Figure 5:**
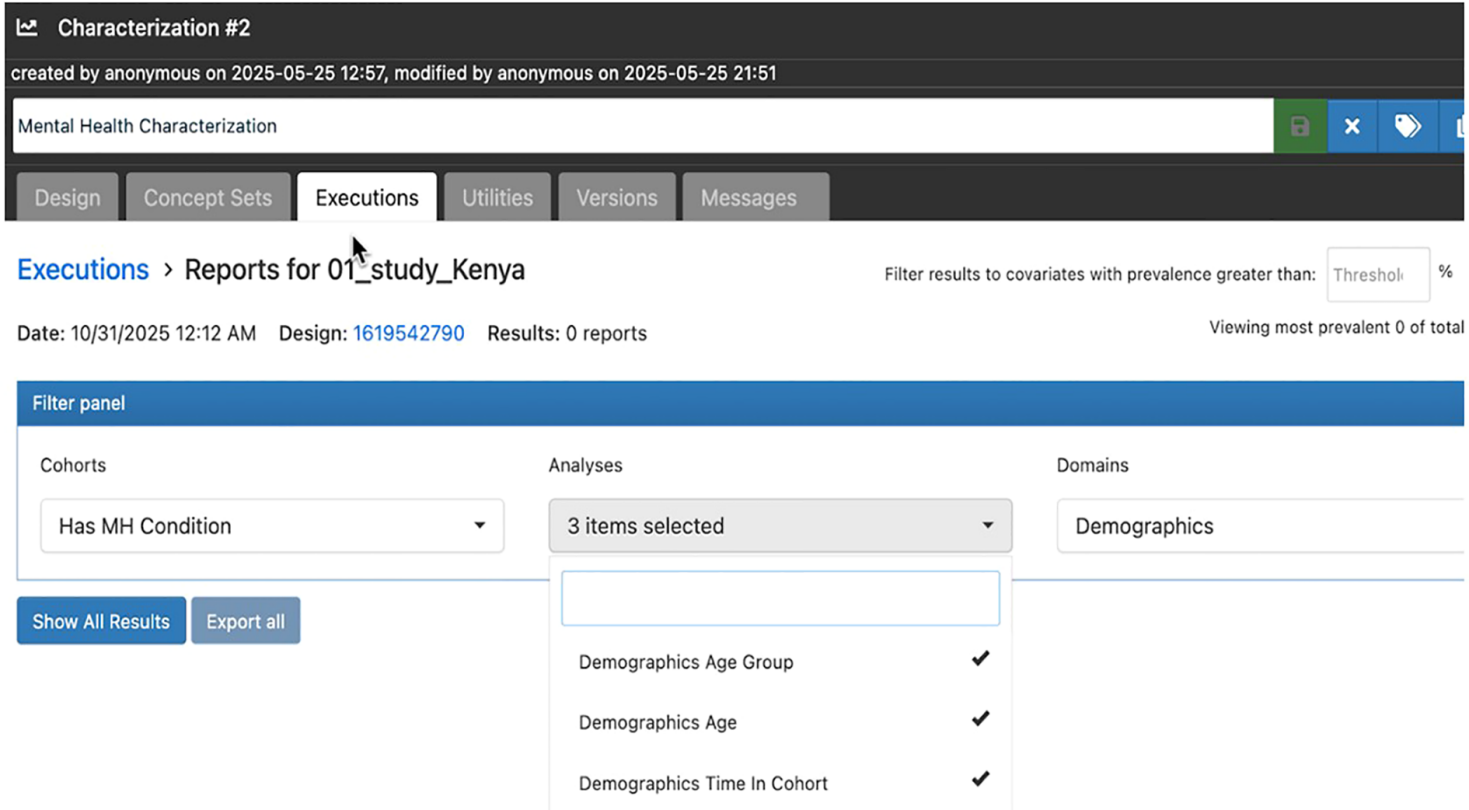
Characterization analysis setup in ATLAS for mental health cohorts.

In Study #1 (study ID = 1), the cohort labeled “has mental health condition” is shown in **Figure 5**, illustrating the ATLAS configuration for demographic analyses and cohort selection, and highlighting the process used to generate descriptive reports for subjects with mental health conditions in the Kenya study.

#### 3.5.1 Incidence analysis

**Figure 6:**
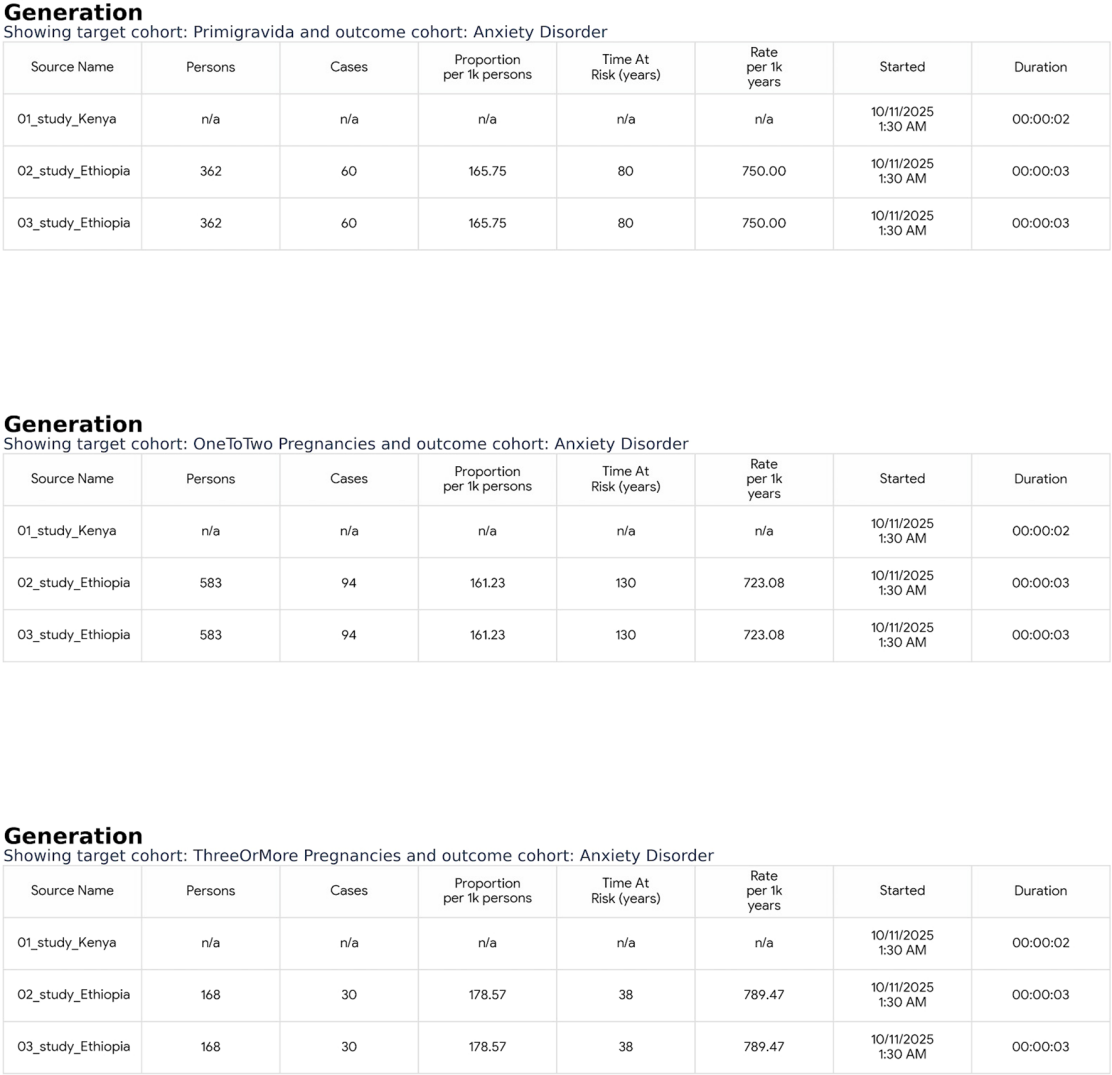
Incidence of anxiety disorder by maternal parity across studies in ATLAS.

Incidence analysis requires cohorts to have data on both the numerator and denominator. In studies #2 and #3, incidence rates for the anxiety disorder outcome were calculated across three parity cohorts (Primagravida, OneToTwo pregnancies, ThreeOrMore pregnancies), where the anxiety disorder outcome is the numerator and different parity cohorts serve as the denominator. See **Figure 6** below. Here, data are shown for Study #1 (Kenya) and Studies #2 and #3 (Ethiopia); the same has been done for the other studies, but not shown in the figure.

For each parity group, the table in **Figure 5** summarises: Number of persons at risk, Number of anxiety disorder cases, Cumulative time at risk (years), number of cases per 1,000 persons, and Incidence rate per 1,000 person-years. Time at risk defines the time window relative to a parity group’s start or end date, with an offset to consider the person ‘at risk’ of the outcome.

In Kenya, data for anxiety disorder among these parity cohorts were not available (n/a). Analysis was conducted using ATLAS for other studies separately. Rates were stratified by the ages of the women in the parity cohorts (not shown here).

#### 3.5.2 Next step of analysis

Across all of these descriptive analyses, we wanted our meta-analysis to surface factors, covariates, or, again, “features” we could explore in future studies that conduct causal analyses like emulated clinical trials and predictive analysis machine learning experiments using OHDSI and other data analysis platforms. New initiatives like Data Science Without Borders (DSWB) and the African Population Cohort Consortium (APCC), both funded by the Wellcome Trust, present these opportunities. In a future paper, we will present the completed results of our meta-analysis together with the bridges we are building between the OHDSI toolset and other research platforms that specialize in predictive analysis, machine learning (AutoML), federation (DataSHIELD), environmental epidemiology (the OHDSI GIS extension), and disease progression modelling (DPM360). In the meantime, this report puts a spotlight on the beginning of that journey, which started with the OHDSI toolchain and the support it provides for the foundational data preparation step called feature engineering.

## 4 Discussion

This study demonstrates how standardisation and integration of African longitudinal mental health data is achievable using the OMOP Common Data Model within the INSPIRE Network Datahub. Central to this success was extensive preparation of the staging database, including substantial manual input, careful coding, and validation, demonstrating that reliable outcomes require harmonised, well-documented source data and transparent, reproducible pipelines. While it cannot simply ingest arbitrary datasets and expect similarly high-quality results, the advances made in vocabulary alignment, metadata standards (DDI Lifecycle), and snowflake schema design have now made the process significantly more straightforward for future data additions.

Importantly, our ETL pipeline and harmonisation workflow produced consistently high-quality data, with over 98% pass rates for conformance, completeness, and plausibility. Automated SQL routines and error logging streamlined missing data management, and regular use of OHDSI quality assurance tools (ACHILLES, Data Quality Dashboard) enabled rapid validation of results. This not only assured data integrity but also saved considerable time in the cleaning and review process, provided someone still checks and interprets the outputs.

The integrated OMOP CDM environment enabled robust descriptive analysis and cohort characterisation, leveraging open-source OHDSI tools such as ATLAS. In this context, cohort construction refers to extracting and manipulating data subsets for analysis, and characterisation means performing descriptive statistics across key domains—including depression and anxiety measures (stress was not specifically measured in this paper). The enhancement here is not in the outputs themselves, but in the fact that researchers can now produce reliable, comparable results with less technical expertise and manual effort than previously required.

While a meta-analysis or formal federation of data sources was not part of this work, our approach lays the foundation for future network-wide observational studies, underpinned by harmonised structures, standard vocabularies, and flexible analysis pipelines. Achieving this required substantial groundwork—good documentation, harmonised data, transparent processes, and the adoption of open-source analytical tools that support both no-code and low-code workflows, making large-scale, collaborative mental health research increasingly practical and sustainable in diverse African contexts.

Unlike prospective harmonisation initiatives, the approach presented here operates entirely on legacy psychiatric datasets collected without a common data model. Compared with prior harmonisation efforts that rely on manual curation or rigid ontology enforcement, our pipeline emphasizes semi-automated vocabulary mapping, combined with study-specific concept extensions to prevent semantic loss. This balance between standardisation and contextual fidelity is particularly important for mental health data collected in low-resource settings, where local instruments and culturally specific constructs are common.

While motivated by psychiatric research questions, the primary contribution of this work lies in health data engineering and retrospective harmonization methodology, with applicability beyond mental health.

## 5 Challenges

Three challenges encountered in this study are particularly noteworthy. First, while the OMOP Common Data Model itself supports analytical interoperability and is designed to enable federated analyses without requiring physical data centralisation, ethical, governance, and consent constraints presented a major practical barrier to pooling sensitive mental health data across sites. Secondly, although all source studies were faithfully represented in the staging database, fundamental differences in study design, methodology, and measurement made it impossible to migrate all studies into a single OMOP CDM instance, which imposed limitations on the subsequent analysis approach. Thirdly, while the development of custom vocabularies improves inclusivity, their long-term utility depends on ongoing curation and global standardisation efforts to ensure the wider applicability and sharing of Africa-specific mental health concepts. Finally, extending this harmonisation framework to other health areas and regions will require careful adaptation and significant additional resource investment.

## 6 Conclusion

This study introduces the design and implementation of a scalable solution for harmonising longitudinal mental health data from Africa using the OMOP Common Data Model in the INSPIRE Network Datahub. By standardising disparate datasets through a metadata-rich staging database, developing domain-specific local vocabularies to bridge the semantic gap, and using ETL pipelines, we have ensured a robust migration of the data with high semantic content. The connection and interoperability with the OHDSI ecosystem support high-performance characterisation, cohort analyses, and cross-study comparisons to advance cross-national mental health research and policies. We are closing the gap between African local data and international/global data standards, which will lead to greater stability, reusable data, and analytical power in mental health research. Our contributions lay the groundwork for future federated studies and evidence-based interventions across the African continent.

## 7 Future steps

Future goals include expanding the INSPIRE Datahub’s reach to include more cohorts and countries, contributing validated local concepts to the global OMOP vocabulary, and testing federated learning approaches for predictive modeling across sites. A wider use of metadata-driven designs and open analytics could change mental health research in low-resource areas, providing richer, more useful evidence for global health policy.

## Data Availability

The raw data supporting the conclusions of this article will be made available by the authors, without undue reservation.
